# Textile Encoding Inspired by Langer Lines via Elastically Graded Embroidered Tessellations

**DOI:** 10.1002/adma.202500959

**Published:** 2025-07-06

**Authors:** Leonid Zinatullin, Mona Küüts, Alvo Aabloo, Indrek Must

**Affiliations:** ^1^ IMS Lab Institute of Technology University of Tartu Nooruse 1 Tartu 50411 Estonia

**Keywords:** bioinspiration, embroidery, hierarchical packing, mechanical metamaterials, skin tension lines, textile robotics

## Abstract

Morphological anisotropies and nonlinear mechanical properties shape the kinematic agility of organisms and engineered structures. Tissues, such as skin, act as biological metamaterials whose internal structures, such as packed collagen fibers, produce nonlinear and directional responses. Similarly, hierarchical thread packing governs the mechanical response of textiles. However, the capability of encoding textiles at the fabric level and at a sufficient resolution remains limited. Embroidering triangularly tiled zigzag patterns of inextensible thread on a stretchable fabric is demonstrated to encode distributed, directional stress–strain behavior at subcentimeter resolution, sufficient to mimic complex physiological characteristics such as Langer lines of the skin. The triangular unit cells solved the Königsberg bridge problem of filling a plane with a continuous thread pair. The encoding maps directionality, directional contrast, and magnitude, three parameters that define unit cells' compliance, into hue‐saturation‐value color space for rapid qualitative design. Cross‐talk‐free arrays of embroidered restrictors enable scalable augmentation of textile mechanics. The transition threshold from the matrix‐ to fiber‐defined behavior is achieved with 85% fidelity, enabling customizable shape‐morphing structures such as restrictor bladder actuators and feet‐conforming shoes. Industry‐standard machine embroidery scales biomechanics‐inspired structures for wearable and environmental robotics and promises future biohybrid technologies.

## Graphical abstract

A standard embroidery machine can encode skin‐like directional mechanics into stretchable fabrics that, when worn, augment the wearer's skin. Zigzag stitch patterns, inspired by skin collagen structure, pack extra slack in threads that, when tessellated, mimic Langer lines. Pneumatic fingers with multiple bending modes and feet‐conforming footwear, designed using intuitive color coding, illustrate uses in robotics.

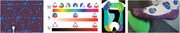

## 1 Introduction

Mechanical coupling in anisotropic and heterogeneous materials can encode complex programmatic distributed behavior, assisting natural and engineered systems in physical interaction with the external environment to achieve the desired actions.^[^ ^1^, ^2^, ^3^ ^]^ For instance, human skin processes internal and external deformation in situ to protect and augment the body. The skin's mechanical behavior is encoded through the strategic packing of collagen fibers in a tessellated matrix of compliant cells. Each local cellular neighborhood processes physical stimuli exerted by surrounding tissue. This local processed response is transmitted among the neighboring cells and propagated to more distant cells. This collective information processing produces tissue‐level properties. In engineering, a similar approach is utilized in mechanical metamaterials, where unit cells are tessellated into large‐scale structures that develop and express emergent properties,^[^ ^4^ ^]^ i.e., properties not exhibited below the unit cell scale, e.g., the negative Poisson ratio.^[^ ^5^, ^6^ ^]^ Therefore, global tissue properties can be encoded by local adjustments in cellular properties. In engineering, mechanical metamaterials with tessellated encoding have been demonstrated to display memory,^[^ ^7^ ^]^ computation,^[^ ^8^ ^]^ and learning.^[^ ^9^ ^]^ However, most metamaterials function only in a controlled environment, are resource‐intensive to produce,^[^ ^9^ ^]^ are difficult to encode, and/or have low input‐to‐output granularity.

**Figure 1:**
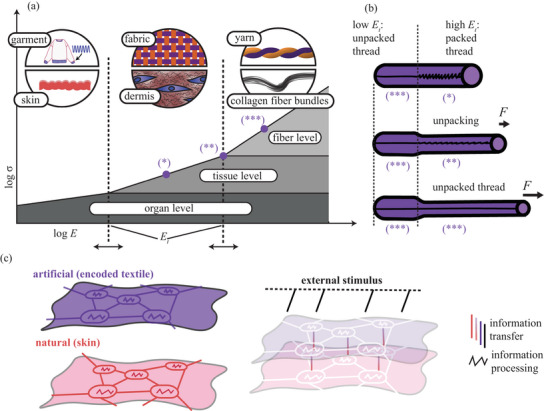
Encoding Via Hierarchical Packing in Natural and Synthetic Systems: Concept. a) Progressive elongation leads to hierarchical unpacking both in natural and synthetic systems, represented by skin and garment, respectively. b) A denser packing shifts the tissue‐fiber transition strain *E*_*t*_ towards larger strain *E* values at applied tensile force *F*. c) Cooperation of frictionally coupled skin and encoded textile as a natural and synthetic, respectively, metamaterial. The collagen fiber bundles (in the skin) and packed and tesselated yarns (in the textile) process mechanical information. The thread loop coupling between unit cells and the frictional coupling between contacting layers transmit information.

Textiles are metamaterials^[^ ^10^ ^]^ that consist of hierarchically packed fibers (high‐aspect‐ratio flexible rods).^[^ ^11^ ^]^ The fibre arrangement pattern^[^ ^12^ ^]^ and packing density determine the mechanical properties of a textile.^[^ ^10^ ^]^ Textiles have an extremely low resistance to coplanar compression while sharply opposing coplanar elongation upon reaching a specific transitional strain. Similarly, in human skin, progressive straightening of collagen fibers produces a J‐shaped stress–strain response.^[^ ^13^ ^]^ At the organ level, the dermal tissue is packed into creases, such as those over finger joints. At the tissue level, the response is encoded by directionally packed collagen fibers in a soft cellular matrix.^[^ ^13^ ^]^ The directional encoding at tissue and fiber levels contribute to Langer lines, a structure that determines the stress‐strain properties as well as tension of the skin.^[^ ^13^ ^]^ Finally, the collagen fibers^[^ ^14^ ^]^ are packed into bundles at the fiber level (**Figure** **1**a). Thus, both skin and textile are examples of hierarchically packed structures that encode behavior via fiber packing density at various hierarchical levels (Figure 1a). Abstracting the evolutionarily validated programming of skin, in particular its fiber‐packing strategy (Figure 1b), could enable new textile encodings that offer finer control over scale‐specific emergent properties, such as those expressed at the organ‐level but not at the tissue or fiber level. A textile worn on the skin and programmed with skin‐inspired methods therefore offers a natural means of augmenting human biomechanics. When layered and frictionally coupled^[^ ^15^ ^]^ with the skin (Figure 1c), it can support the expression of organ‐level properties. Additionally, standalone skin‐inspired technologies are enabled. While actively controlling membrane stiffness patterns^[^ ^16^ ^]^ promises versatile adaptation across tasks and even gait phases, informed design of passive wearables can also significantly reduce metabolic cost.^[^ ^17^ ^]^ However, current passive encoding solutions capture only a fraction of biomechanical complexity, particularly in terms of encoding resolution, anatomical specificity (e.g., informed of or aligned with Langer lines), and task context. Encoded designs are also more robust than systems that rely on sensor‐based feedback loops. However, as also noted by Chellattoan and Lubineau, stretchable membranes with high stiffness contrast are extremely challenging to achieve.^[^ ^16^ ^]^

The choice of the hierarchical level for textile encoding^[^ ^11^ ^]^ has several practical tradeoffs. Global stitching, where prefabricated anisotropic fabric pieces are sewn together in a specific orientation and pattern, is equivalent to organ‐level programming (Figure 1a). The individual mechanical responses of the fabric pieces define the global stress–strain behavior of the sewn piece. For instance, the morphology of high‐altitude parachutes at varying dynamic pressure is achieved by global stitch encoding.^[^ ^18^ ^]^ Although established, this method requires artisanal labor and is therefore limited in unit cell count and density. At the other end of the scale – fiber‐level encoding^[^ ^19^ ^]^ necessitates complex treatments and highly specialized equipment. Therefore, the intermediate—tissue—level appears most promising for textile encoding.

At the fabric (corresponding to tissue) level, material encoding by machine weaving and knitting^[^ ^20^, ^21^, ^22^ ^]^ offers good spatial resolution, is fast, and enables non‐Euclidean surfaces. However, arbitrary‐pattern machine knitting requires complex machinery. It produces material that has inherently different properties in weft and warp directions while also being difficult to set up and design for. The machine‐knit encoding is inherently discrete due to a finite number of thread loop combinations, disallowing continuous adjustment of mechanical properties.

Machine embroidery allows for arbitrary thread patterns, but is comparatively less explored for encoding material anisotropies, potentially due to opinions regarding the complexity of working with stretchable substrates.^[^ ^23^ ^]^ Machine embroidery lays down thread pairs, one on either side of the substrate. At every stitch, the substrate is punctured and the two threads are twisted together. As a result, the embroidery machine can attach the thread pair to a flat sheet at an arbitrary location in an arbitrary order. The ability to embroider arbitrary fiber packing patterns and densities enables localized and granular mechanical behavior to be encoded. Kiourti et al. have demonstrated embroideries approaching thread thickness (0.3 mm) in resolution.^[^ ^24^ ^]^ Another benefit of machine embroidery is that it can encode isotropic materials to produce well‐defined material anisotropies, e.g., Ceron et al.^[^ ^25^ ^]^ used embroidery to encode spiral behavior into a flat sheet of elastomer. Guo et al.^[^ ^26^ ^]^ demonstrated encoding 1D targeted deformation into restrictor bladder actuators by varying the zig‐zag stitch density in sewing. Since sewing is high‐aspect‐ratio embroidery, thread packing by embroidery emerges as a scalable encoding method.

This work expands the concept of hierarchical fiber packing into a cost‐effective and resilient tessellated skin‐like encoding strategy. We present a skin‐like textile mechanical metamaterial that can be cost‐effectively designed and customized using an intuitive color space representation, can be mass‐produced, is resilient enough to wear, and has non‐discretized unit cell encoding. The encoding was carried out on an anisotropic, compliant knit fabric. Encoding fidelity was demonstrated via tensiometry, the emergence of global behavior by local encoding was demonstrated via encoding restrictor bladder actuators, and resilience and scalability were demonstrated via conformable footwear.

## 2 Results and Discussions

### 2.1 Packing by Embroidery

In this work, an elastic membrane is encoded at the hierarchical packing level corresponding to the tissue in the skin. Similarly to the skin, the mechanical properties are programmed by locally and directionally encoding the transitional strain *E*_*t*_ at which the material transitions from tissue‐defined behavior to fiber‐defined behavior (Figure 1a). As with collagen in the skin, an elastic fabric is encoded by strategically adding packed restrictor fibers. The fibers were machine‐embroidered on the fabric.

The ratio between the length of an inelastic thread *l* *′*, packed into a linear segment of length *l*, is referred to as linear fiber packing density *µ* as

(1) $$\mu \equiv \frac{l^{\prime }}{l}$$

The packed inelastic fiber stretched to the limit develops stretch *λ* ^*max*^ numerically equal to *µ* as

(2) $$\lambda^{max}\equiv \frac{l+\Delta l^{max}}{l}=\frac{l^{\prime }}{l}=\mu$$

As fibers are 1D, a high *µ* is easily achievable on a plane. The work envelope of the encoded structure is limited by the properties of the elastic membrane and its interaction with the encoding. As maximum strain *E* is expected at *λ* ^*max*^, and *λ* ^*max*^ corresponds one‐to‐one to *µ*, we approximated *E*_*t*_ to be a fixed fraction of *µ*. Henceforth, the proportionality constant *α* connects *µ* and *E*_*t*_ as

(3) $$E_{t}+1=\alpha \left(\mu +const\right)$$

**Figure 2:**
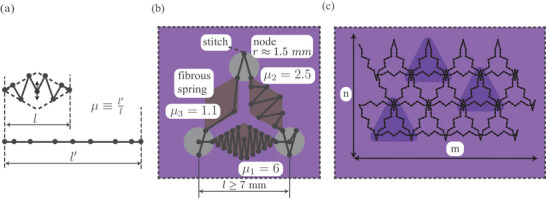
Hierarchically Tesselated Fiber Packing: Concept. a) A rhomboid fibrous spring with continuously programmable *E*_*t*_. b) A triangular unit cell enables singularly defined planar anisotropic stress–strain behavior. c) Unit cells tessellated into a tissue‐level‐programmed textile metamaterial.

In the interest of tilability, the threads were arranged into 60 − 120° equally‐sized rhombi. Embroidery produces a sequence of straight dual‐thread segments intersperced by stitches. The most effective way of packing the fiber is, therefore, by amplitude‐modulated zig‐zag. The number of undulations changes *µ* in discrete steps, whereas the fine adjustment is achieved by modulating the amplitude of the largest (i.e., the middle) undulation (**Figure** **2**a). The resulting pattern, shown in Figure 2a, in combination with a soft matrix, is the smallest unit of the encoding strategy, a ‘fibrous spring.’ A fibrous spring with *µ* = 1 is unpacked, i.e., consists of colinear thread segments. Three fibrous springs arranged into a triangle constitute a unit cell, as shown in Figure 2b. As a triangle is singularly defined by the lengths of its sides, the three *µ*‐s singularly define the directional force‐strain response of a unit cell. The unit cells are, in turn, tiled into a packed fiber array. The force‐strain response of the array is encoded using the packing tensor *P*_*hij*_ with dimensionality [*m*, *n*, 3], where there are *m* × *n* unit cells, each containing three fibrous springs (Figure 2c).

### 2.2 Raster Graphics Representation

**Figure 3:**
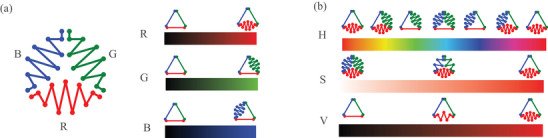
Color Space Representation of Packing Tensor a) RGB color space. Pixel channel values map to *µ*‐s in a unit cell. b) In HSV color space, hue (H) corresponds to directionality, saturation (S) corresponds to directionality contrast, and value (V) corresponds to direction‐independent stretchability magnitude.

The dimensionality of the packing tensor is equal to the dimensionality of an RGB image, which allows for direct mapping of channel values of an image to the packing tensor (and vice versa), as shown in **Figure** **3**a. This mapping enables the use of industry‐standard image manipulation tools in the design process of the packed fiber arrays, enhancing thereby the scalability and approachability of the encoding process. Furthermore, it is useful to leverage HSV (hue, saturation, value) color space, which is, in effect, the polar coordinate system version of the RGB color space. As shown in Figure 3b, hue represents the directionality of a unit cell, saturation denotes the directional contrast, i.e., unidirectional versus omnidirectional stretchability, and value represents the direction‐independent stretchability magnitude.

### 2.3 Königsberg Bridge Problem and the Interconnects

**Figure 4:**
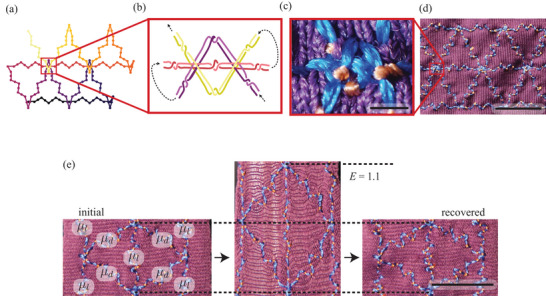
Single‐Line Tesselation of a Thread Duplet. a) A solution to the Königsberg Bridge Problem; b) schematic of an interconnect between adjacent unit cells; c) photograph of an interconnect; d) photograph of a completed array. e) Reversible deformation of an encoded sample. A sample encoded in *µ*_*l*_ and *µ*_*d*_ directions shown as‐fabricated (left), stretched in *µ*_*l*_ direction (center), and recovered (right). Horizontal dashed lines are guides for the eye. Scale bars: c) – 2 mm, d, e) – 10 mm.

Tissue‐level behavior necessitates communication between unit cells, achieved via robust coupling. The intersections between adjacent unit cells transmit force information between fibrous springs that process this information, recalling Figure 1c above. Sewing and embroidery effectively position 1D threads on a plane, implying a different set of design rules compared to other additive manufacturing techniques, such as fused deposition modelling. Keeping the fibrous array from unraveling requires the entire array to be manufactured using a single continuous pair of threads. Embroidery constraints do not support interconnecting adjacent fibrous springs in a node by recurring stitching to the same point. Additionally, each fibrous spring can be embroidered once. This presents a manufacturing challenge for the packed array, as every connecting node (except for the first and the last one) has to have an equal number of non‐repeating inbound and outbound paths. This constraint is known as Königsberg Bridge Problem (crossing all bridges by walking each bridge once). This constraint is satisfied by embroidering the encoding in the order presented in **Figure** **4**a. Unit cells were coupled by arranging stitches in the nodes to always form thread loops around the previously deposited threads (further preventing unravelling), as conceptualized in Figures 2c and 4b and implemented in Figure 4c.

### 2.4 Integrity of Encoded Embroidery

Our encoding is reliable: in processing roughly 175 different encodings (including batch production), some composed of more than a thousand unit cells and tens of thousands of stitches, the encountered defects were due to human error (e.g., the supporting thread ran out). After manufacture, the samples withstood multiple (tens to hundreds of) elongations past *E*_*t*_ with only minor temporary viscoelastic residual strain. Figure 4e shows residual strain as localized fabric lengthening, which appears as small wrinkles.

### 2.5 Encoding Fidelity

**Figure 5:**
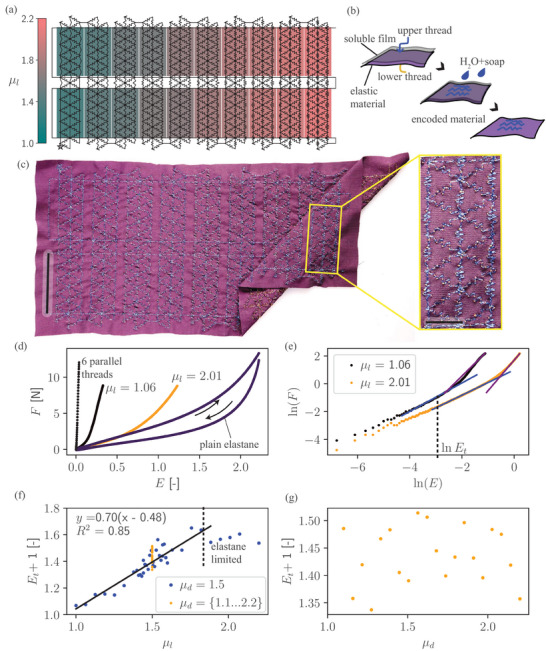
Fidelity of the Embroidered Encoding. a) Embroidery pattern for *µ*_*l*_ sweep b) Principle of embroidery on an elastic substrate. c) Batch embroidery of tensiometric samples for *µ*_*l*_ sweep. d) Representative force‐strain plots of elastane encoded at small and large *µ*_*l*_, compared to non‐encoded substrate and free‐standing encoding threads. All curves, except for the plain elastane, are from unidirectional pull. e) Representative tensometric results in log–log scale, showing distinct linear behavior at corresponding hierarchical packing levels. The inflection point corresponds to the programmable tissue‐fiber *E*_*t*_. f) The sweep of *µ*_*l*_ shows high‐fidelity programmability. g) Sweep of *µ*_*d*_ confirming directional independence. Scale bar: c) – 45 mm (left) and 10 mm (right).

We encoded the local tensiometric response by defining *E*_*t*_ at the unit cell level. The dependence of *E*_*t*_ on unit cell parameters was characterized by sweeping *µ* in both the pulling direction (*µ*_*l*_) and the ±60° diagonal direction (*µ*_*d*_). **Figure** **5**a exemplifies a batch of 20 tensiometric samples, with a parameter sweep in *µ*_*l*_. The manufacturing process detailed in Figure 5b results in the encoding presented in Figure 5c. The samples were later separated in preparation for tensiometry. This approach enables a scalable mass manufacturing strategy of encoded structures using industry‐standard equipment.

Figure 5d exemplifies force‐strain responses of the samples with a large and a small *µ*_*l*_ (2.01 and 1.06, respectively). At small elongations (*E* ≪ *E*_*t*_), the force‐strain response of the encoded textile closely matches the plain elastane substrate. At the ultimate elongation (corresponding to *λ* ^*max*^), the tensile samples converge toward a response similar to that of six parallel threads (three pairs of threads that make up three parallel rows of fibrous springs). These two extremes correspond to two packing ranks (tissue‐ and fiber‐level, respectively), and adding packed restrictor fibers results in a stress–strain response, which is between these extremes. Although elastane exhibited a noticeable hysteresis in its force‐strain response (Figure 5d), it did not affect *E*_*t*_ as the root cause was the strain‐rate‐dependent properties of elastane, which affect the dynamics of the system and not the geometry of the unpacking fiber. Figure S1 (Supporting Information) confirms that energy retention of the encoded fabric comprises comparable contributions from a rate‐dependent component, arising from viscoelastic material behavior, and a rate‐independent component, evidence of frictional coupling between crossing threads. Together, viscoelasticity and friction constitute a form of volatile memory with recovery times determined by the fabric properties. In a simplified depiction, the encoded fabric dissipated approximately 20% more energy during the first cycle following a one‐hour rest, as demonstrated in Figure S2, (Supporting Information).

The two hierarchical packing regions of the encoding are most apparent in a log–log plot, exemplified in Figure 5e, where there are two distinct linear regions separated by a transition region. The inflection point was found via segmented linear regression and plotted against *E*_*t*_ in Figure 5f. The largest practical *µ*_*l*_ with the given fabric was *µ* = 1.8, which is explained by the substrate material properties: as seen in Figure 5d: the substrate fabric itself transitions to the fiber defined behavior around *µ* = 0.8…1.2. Fitting linear regression to the points before *µ*_*l*_ = 1.8 resulted in *R* ^2^ = 0.85, meaning the encoding was 85% effective (85% of the variance in *E*_*t*_ was explained by variance in *µ*_*l*_). Doing the same for *µ*_*d*_, aggregated in Figure 5g, did not show any trend with *R* ^2^ = 0.002, implying each fibrous spring within a unit cell has independent control over the directional response of the material. The remaining ∼15% of the variance may be attributed to manufacturing tolerances, the inherent variance of properties of the materials, measurement error, and limitations of the segmented linear regression model.

The fitted strain‐referenced proportionality constant *α*_*E*_ = 0.70 in *E*_*t*_ ∝ *µ*_*l*_ (Figure 5f) quantifies the influence of fibre constraint pattern on system‐level stiffness. Reverse‐scan fit yielded an even higher *α*_*E*_ = 0.85, as shown in Figure S3 (Supporting Information), evidencing the memory effect due to viscoelasticity and friction. While averaging *E* over the entire restrictor‐membrane system, as in Figure 5d–g, is practical for metamaterial analysis, we conducted image‐based stretch mapping of a fully extended (*λ* = 1.55) fibrous spring (*µ*_*l*_ = 1.55) to resolve individual stress concentration hotspots in the knit membrane. As reported in Figure S4 (Supporting Information), local stretch reached *λ* = 2.2 at the center of the fibrous spring, where cross‐stitch stress localizations are expected, while the knot regions developed significantly lower values (approximately *λ* = 1.2). The discrete structure of the knit membrane expectedly led to pronounced local fluctuations in *λ*. Moreover, Figure S4 (Supporting Information) shows the system was stretched to *λ* = 1.66, slightly exceeding the *λ* = 1.55 threshold for a fully unpacked fibrous spring. This suggests that the tissue‐level stretch capacity was exhausted, activating a deeper hierarchical level of fibers: each stitch loops the pair of threads and twists them into a helical architecture that allows additional stretch.

Alternatively, we proposed stretch‐referenced proportionality constant *α*_*λ*_, defined by the relation *λ*_*t*_ ∝ *µ*_*l*_. The fitted value *α*_*λ*_ = 0.59 is presented in Figure S5 (Supporting Information). The introduction of *α*_*λ*_ enabled us to also define the ratiometric unpacking parameter *λ*_*t*_/*µ*_*l*_ that provides an informative and intuitive representation of fibrous spring unpacking: at small *µ*_*l*_, only the fiber is engaged, whereas at large *µ*_*l*_, the encoding engages when the system is stretched to 59% of its maximum stretch capacity (*λ* = *µ*_*l*_) for the given membrane.

To test the pattern specificity of *α*_*E*_, we analyzed a fibrous spring constructed with rectangular, rather than zigzag, primitives (Figure S6, Supporting Information). The rectangular pattern yielded a lower fitted *α*_*E*_ = 0.66, suggesting that the local stretch entered the nonlinear region of the *E*‐*F* curve of the membrane (see plain elastane in Figure 5d) at a lower global stretch due to more pronounced cross‐stitch stress and stretch localizations. As expected, the increased stretch granularity of the rectangular encoding reduced the fit quality, with *R* ^2^ = 0.74.

### 2.6 Local Encoding Determining Global Behavior in Pneumatic Restrictor Bladder Actuators

In biological systems, global emergent properties are determined by the aggregated properties of tissue subunits.^[^ ^27^ ^]^ This principle also informs synthetic designs, such as the restrictor bladder actuator, where the local stress–strain responses of the restrictor determine the global shape of the actuator in response to internal (relative to atmospheric) gas pressure *p* and external mechanical stimulus. We demonstrate that programming local and directional *E*_*t*_ produces four superimposable global actuation patterns: ‘bending,’ ‘twisting,’ ‘elongation,’ and ‘no movement.’

**Figure 6:**
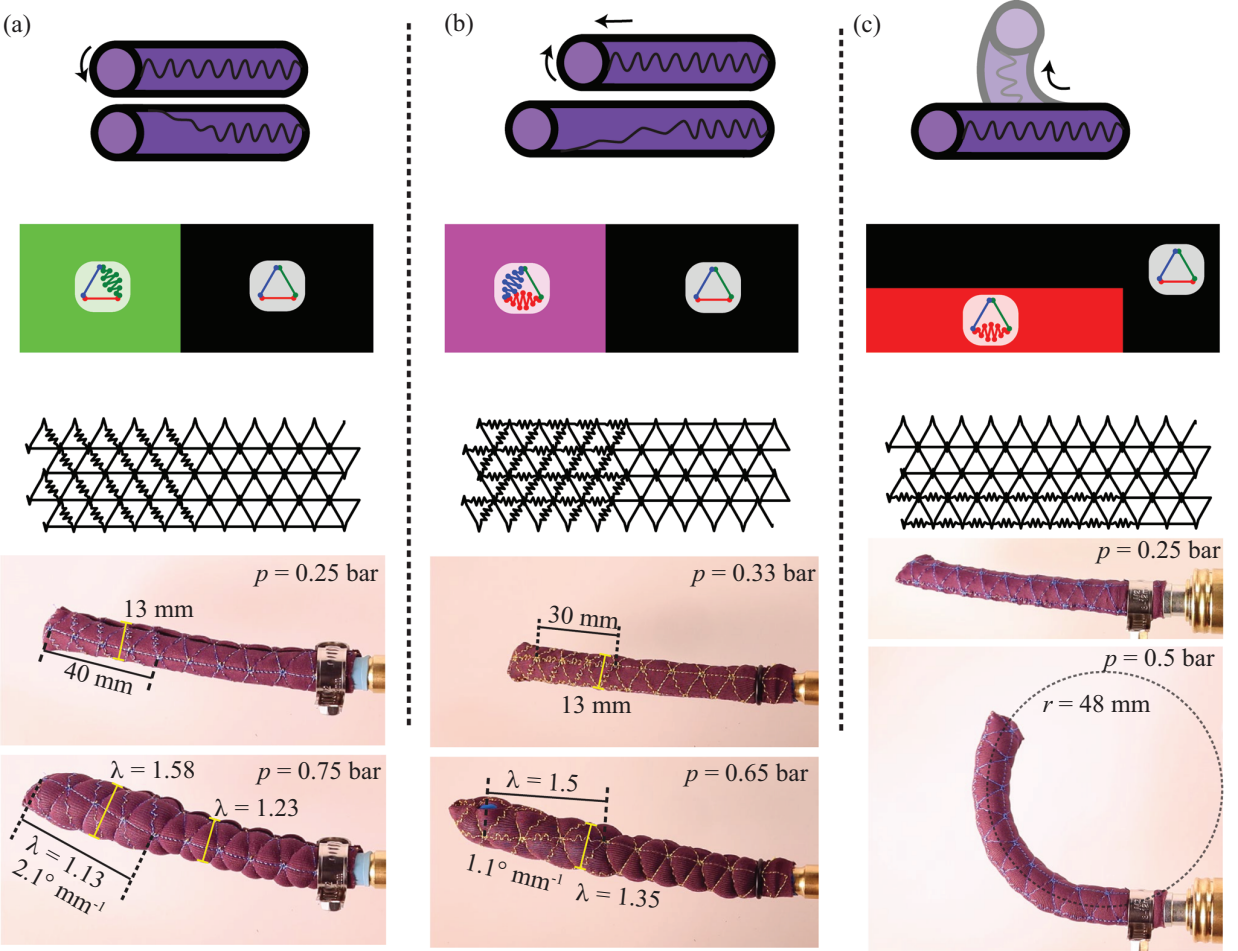
Local Encoding Determines Global Behavior of Restrictor Bladder Actuators. a) The distal half of the actuator programmed to twist, while the basal region was encoded for minimal deformation. b) The distal half of the actuator programmed to twist and elongate. c) The actuator encoded for uniform bending. In all panels, images proceed from top to bottom: desired deformation mode, HSV color map, encoding pattern, and demonstration.

Twisting motion was induced by spirally encoding regions of higher *µ*, as shown in **Figure** **6**a. This encoding pattern constrained axial elongation, resulting in pronounced twisting (1.8° mm^−1^) with little elongation (*E* = 0.17). The elongation was superimposed on twisting by adding a higher *µ* in the axial direction. As shown in Figure 6b, this resulted in a higher elongation of *E* = 0.39 at the cost of a lower twist (0.5° mm^−1^). A basal section of the actuators shown in Figure 6a,b was further omnidirectionally encoded with *µ* = 1, rendering the corresponding section inert. The bending motion was achieved by asymmetrically encoding fibrous springs in the axial direction. As shown in Figure 6c, the convex side of the restrictor had a higher linear fiber packing density (*µ* = 2.3), leading to a higher *E*_*t*_, which, upon inflation, resulted in differential elongation and bending motion. As seen in the Movie S1 (Supporting Information), the movement of the pneumatic actuators was rapid and limited not by the viscoelasticity of the fabric but by the pressurization and depressurization rate of the bladder.

As in human skin, encoding local properties determines global behavior. Our approach is intuitive, as the ultimate stretch *λ* ^*max*^ of the spring is directly proportional to *µ* (Figure 5f) and adjacent springs encode the tissue independently (Figure 5g). To further demonstrate the programmable nature of transitional strains, we explored a trimodal actuator where deformation modes (elongation, twist, and bend) were accessed sequentially with increasing internal pressure (Movie S2 and Figure S7, Supporting Information). Lengthwise fibrous springs asymmetrically encoded at *µ* = 1.6 and *µ* = 1.3 enabled elongation and later local bending. At moderate pressure, diagonal threads encoded at *µ* = 1.3 unpacked, while the opposing diagonal threads at *µ* = 1.0 remained stiff, causing the finger to twist clockwise and elongate. When the fibrous springs at *µ* = 1.3 crossed *E*_*t*_, the finger started to bend due to the remaining packed length in the *µ* = 1.6 thread, creating a local curvature that translated into a global helix due to the earlier twist. This sequence illustrates how spatially distributed transitional strains can passively encode temporal deformation modes—a capability not available in the cylindrically wound restrictors of McKibben‐type effectors and currently achievable only through active restrictor clutching mechanisms.

### 2.7 Wearable Implementation and Scalability Demonstration

Clothing augments human biomechanics—specifically, its textile components in contact with the skin complement the skin's directional mechanical properties, as defined by Langer lines. For example, local and directional support of the footwear prevents the risk of trauma and increases biomechanical efficiency.^[^ ^17^ ^]^ The scalability of machine embroidery promises affordable and tailored biomechanical enhancement by incorporating principles drawn from the very structures it engages, while allowing precise adjustment to individual bodies.

**Figure 7:**
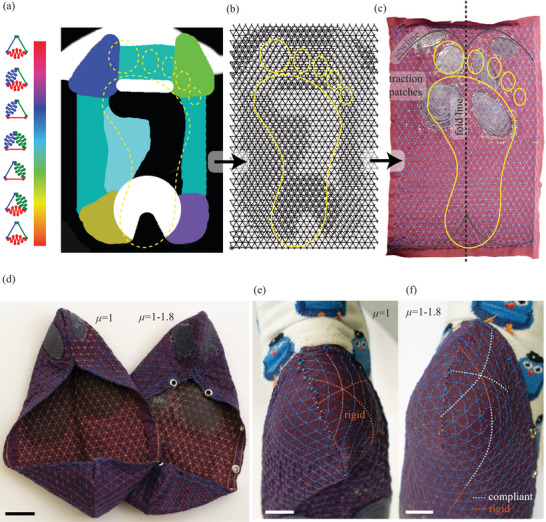
Wearable implementation and scalability demonstration. a) Simplified surface map of human foot skin stretchability with directional *µ* mapped to HSV color space. The yellow line represents the footprint outline. b) Embroidery pattern produced by sampling the stretchability map. c) An encoded fabric as a shoe preform. d) Final shoe with variable encoding (*µ* = 1 − 1.8, right) and a control shoe with uniform encoding (*µ* = 1, left). e,f) Heel regions of the control and variably encoded shoes, respectively, worn by the First Author. The control shoe (e) showed poor fit, while the variably encoded shoe (f) conformed closely to the wearer's heel. Dashed orange and red lines, overlaid on colinear fibrous springs radiating from a reference knot at the heel, indicate rigid and compliant sections. Scale bars: (d‐f) – 20 mm.Wearable implementation and scalability demonstration. a) Simplified surface map of human foot skin stretchability with directional *µ* mapped to HSV color space. The yellow line represents the footprint outline. b) Embroidery pattern produced by sampling the stretchability map. c) An encoded fabric as a shoe preform. d) Final shoe with variable encoding (*µ* = 1 − 1.8, right) and a control shoe with uniform encoding (*µ* = 1, left). e,f) Heel regions of the control and variably encoded shoes, respectively, worn by the First Author. The control shoe (e) showed poor fit, while the variably encoded shoe (f) conformed closely to the wearer's heel. Dashed orange and red lines, overlaid on colinear fibrous springs radiating from a reference knot at the heel, indicate rigid and compliant sections. Scale bars: (d‐f) – 20 mm.

We demonstrate the scalability of embroidery encoding on footwear. First, we created a simplified surface map of human foot skin stretchability inspired by Langer lines (**Figure** **7**a). The pattern was modified to increase the desired local and directional strain limits, such as for increased support in the toe region, while allowing for bending. The design was sketched using digital drawing software in the HSV color space that fully defined local anisotropic stretchability. The surface map image was sampled at a 7‐mm resolution corresponding to *l* of fiber springs and scaled to *µ* = 1 − 1.8. The packing tensor was converted to a 460‐cm^2^ surface map of a matching shoe, featuring 1008 unit cells and 19027 stitches, shown in Figure 7b. A 1000‐fold count of unit cells sufficed to replicate the essential anisotropic strain responses of foot skin. The machine embroidery of the foot surface map on stretchable fabric, shown in Figure 7c, took approximately one hour without human intervention. Finally, the encoded fabric was amended with traction patches, folded, and sewn to produce a shoe (Figure 7d, right). A second shoe uniformly encoded at *µ* = 1 was prepared as a control (Figure 7d, left). The stretch‐insensitized control shoe expectedly showed inferior foot compliance and support, especially pronounced as a poorly fitted heel region in Figure 7e. The encoded shoe shown in Figure 7f closely fitted the foot, including the heel region. Upon testing the footwear, the First Author reported that the encoded shoe supported the foot by preventing toe torsion without restricting flexion, illustrating potential for wearable technologies. The encoded shoe did not develop observable damage even when considerable forces were put upon the shoe upon testing in a climbing park, confirming the resilience of our metamaterial.

Biomechanics‐inspired and ‐informed encoding can be applied to footwear in existing machine embroidery factories at a low investment cost. Machine embroidery accepts a custom pattern for each run, promising rapid customization—our prototype climbing shoes took approximately eight man‐hours from feet measurement to testing. Embroidery encoding preserves economies of scale and provides business opportunities within and beyond orthopaedics.

## 3 Conclusion

Morphological anisotropies enable distributed input–output mappings, producing complex physical behavior and thereby embodying deterministic physical information processing. In mechanical metamaterials—such as the encoded textile described above—each unit cell processes net forces from both the environment and neighboring cells. Such a distributed arrangement is architecturally analogous to a recurrent neural network. Nonlinear activation functions enable such networks to produce complex and distinct behaviors.^[^ ^28^ ^]^

In the demonstrated restrictor bladder actuators, the tension force along any given path on the surface was constant. The stored energy was, therefore, larger in locations with greater expansion (*dU* = σ*dl*). Using variable fiber packing densities, the restrictor directed the work of the expanding gas to act only on the locations (at higher packing density) necessary for the desired actuation pattern. A higher packing density thus leads to a higher local energy density. Above *E*_*t*_, the encoded surface is locally passivated, limiting further expansion. As an expression of a nonlinear activation function, the restrictor develops a defined and continuously adjustable local expansion range, not attainable with a linear system. This local behavior translates into global actuation patterns defined by the packing tensor, input pressure, and environment interactions. Nonlinear restrictor unit cell patterns, encoded at the tissue level, can encode behavior that emerges at the organ level—one level above and absent at the level where it was encoded—in a controlled manner.

The Langer lines of human skin, essentially natural nonlinear arrays of packed collagen fibers, were augmented using a synthetic nonlinear packed fiber array in the shape of footwear. The encoded shoe in contact with human skin demonstrated cooperation between two coupled systems (similar to neural networks, as discussed above) sharing encoding principles. The synthetic system contributed by preprocessing the environmental input before passing it on to the Langer lines. This augmentation could, in perspective, enhance foot mechanics by reducing muscular stress and lowering the risk of injury. A similar principle may be utilized in other garments, such as gloves and bras, as well as wearables like augmented reality goggles and smartwatches. In clinical applications, encoded arrays can act as programmable scaffolds for skin grafts and reconstructive surgeries,^[^ ^29^ ^]^ directly augmenting or replicating Langer lines, thus improving medical outcomes. Skin‐inspired tissue‐level encoding also inspires the design of biohybrids.^[^ ^30^ ^]^

This encoding strategy enables inflatable support structures with customizable Gaussian curvature. When scaled up using roll‐to‐roll embroidery machines (or repurposed computerized quilting machines), non‐Euclidean restrictors can be used, e.g., in scaffolding smart materials^[^ ^31^ ^]^ and construction as temporary inflatable support structures. This would offer a higher degree of customizability, e.g., in the design of concrete domes.^[^ ^32^ ^]^

Both natural and engineered systems structure materials to encode complex behaviors into physical agents. Since clothing is perceived as an extension of the body, encoded textiles could shift wearable devices from perceived as foreign objects to integral bodily components, increasing both physical and social compliance with technology.

## 4 Experimental Section

### Embroidery

Embroidery stitch coordinates were generated using a custom Python library provided in Code Library S1, (Supporting Information). The library fills closed contours with a tiled stitch pattern row‐by‐row according to the packing tensor as input. The pattern was treated as a sequence of stitch coordinates. The top function *triangle_fabric()* transforms the packing tensor into a sequence of fibre springs. The function *spring()* produces fibrous spring shapes according to *µ*, defining local *E*_*t*_. It could do so by utilizing different spring pattern generators such as *spring_zigzag()*, for the default zig–zag pattern, or *spring_alt()*, for an alternative, rectangular pattern. The helper functions *floor_right()* and *roof_left()* append fibre springs, producing non‐unravelling fibrous knots at fibre spring interconnects between rows, as in Figure 4a,b. Each of these knots interconnects an even number of fibrous springs, satisfying the constraints imposed by the Königsberg bridge problem. The function *point_distance_equalizer()* equalizes the spacing between the stitches to ensure optimal stitch length and to satisfy embroidery constraints such as minimum (or maximum) stitch spacing and compensating thread tensioning. The function *anchor()* initiates the stitch sequence to connect the top and bottom threads and prevent the pattern from unravelling. The functions *testfabric()* and *sample_grid()* produce parametrically graded test patterns and arrange them for production, respectively.

Embroidery was performed using a Pfaff Creative Icon machine equipped with an automatic thread tensioning system. The embroidery pattern was compiled into an instrument‐specific binary instruction file using a custom library in Code Library S2, (Supporting Information). Embroidered was performed using Ariadna Fabryka Nici IRIS 40N multifilament polyester thread on a knit elastane (180 g m^−2^ areal weight, 2.1 mm^−1^ line density, Abakhan Fabrics) stacked with a water‐soluble film (50 µm Karnaluks). After embroidery, the film was dissolved using warm (≈35 °C) soapy water. Finally, the samples were dried and optionally separated from a batch.

### Tensometry

Encoded textile samples were made in batches of 20 (Figure 5a,c). Tensometry was carried out in an uniaxial loading configuration using a custom tensile test machine consisting of a vertical leadscrew linear stage and a Wheatstone bridge load cell. The stage was controlled, and data was collected using an ESP32 microcontroller. The sample was strained at 2.2 mm s^−1^. Green strain was used for strain representation as $E=\frac{1}{2}\frac{{\left(l+\Delta l\right)}^{2}-l^{2}}{l^{2}}$.

### Restrictor Bladder Actuators

Restrictor bladder actuators were manufactured by embroidering a fibrous spring array on an elastane substrate using a method detailed in Figure 5b. The fabric was then sewn into a cylinder and capped at one end. A high‐aspect‐ratio (≈6 mm diameter) balloon was placed inside and cut to length. The assembly was then attached to a standard pneumatic fitting and pressurized with compressed air.

### Morphometry

Morphometry was performed by digital planimetry on macro photographs using Adobe Illustrator.

### Footwear

A piece of sacrificial fabric was wrapped around a foot to mark orientations aligned with Langer lines. The fabric was then unwrapped and photographed to produce an approximate foot net. The Langer line directionality on this net was used as a guide to produce encoding of the pattern in HSV color space using Adobe Photoshop. The HSV‐encoded pattern was then exported in RGB format and sampled using a Python script to produce an embroidery pattern with *µ* values ranging from 1 to 1.8. The encoded fabric was sewn into a sock‐like geometry by folding the textile in half and stitching along three sides, leaving one edge open to accommodate the foot. Silicone patches were added to strategic locations (using fabric impregnation) to provide traction. A second shoe was made using the same method but encoded with a constant *µ* = 1.

## Conflict of Interest

The authors declare no conflict of interest.

## Author Contributions

L.Z. and I.M. were responsible for the conceptualization of the study. Data curation and formal analysis were carried out by L.Z. Funding was acquired by I.M. and A.A. The investigation and methodology were conducted by L.Z. and M.K. Project administration was managed by I.M., while software development was undertaken by L.Z. and M.K. Supervision was provided by I.M. and M.K. Validation and visualization were performed by L.Z. and I.M. The original draft was prepared by L.Z. and I.M., who also contributed to the review and editing of the manuscript.

## Supporting information

Supporting Information

Supplemental Movie 1

Supplemental Movie 2

Code‐Library1.py

Code‐Library1.py

## Data Availability

The data that support the findings of this study are available from the corresponding authors upon reasonable request.
