# Supplementary material for: Textile Encoding Inspired by Langer Lines via Elastically Graded Embroidered Tessellations
Source: Adv Mater. 2025 Jul 6;37(37):2500959. doi: 10.1002/adma.202500959 (PMC12447055; doi:10.1002/adma.202500959)
Supplement: Supplementary file 1 — Supporting Information [file ADMA-37-2500959-s001.pdf]

ADVANCED MATERIALS

Supporting Information

for *Adv. Mater.*, DOI 10.1002/adma.202500959

Textile Encoding Inspired by Langer Lines via Elastically Graded Embroidered Tessellations

Leonid Zinatullin, Mona Küüts, Alvo Aabloo and Indrek Must**

Supporting Information for *Textile Encoding Inspired by Langer Lines via Elastically Graded Embroidered Tessellations*

Leonid Zinatullin, Mona Küüts, Alvo Aabloo, Indrek Must**

IMS Lab, Institute of Technology, University of Tartu, Nooruse 1, 50411 Tartu, Estonia

This file includes:

- Supporting Information Figures S1 to S7 with captions
- Captions for Supporting Information Movies S1 and S2
- Captions for Supporting Information Code Libraries S1 and S2

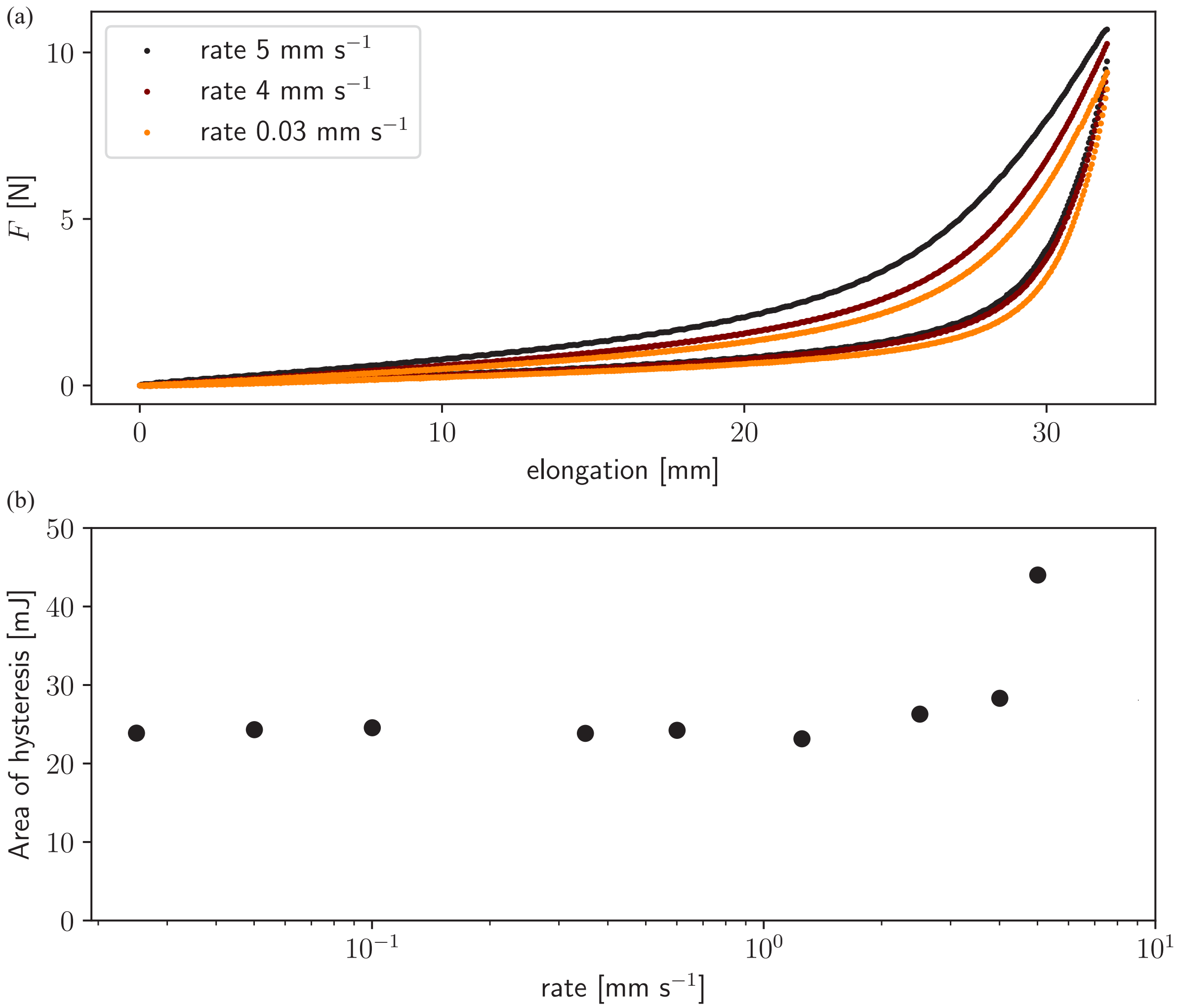

Figure S1: Energy Retention of the Encoded Fabric. a) Hysteresis plots of a sample encoded at $\mu_l = 1.55$ elongated by $l' - l = 32$ mm at strain rates ranging from 5 to 0.03 mm s^{-1}. b) Hysteresis area with respect to elongation rate.

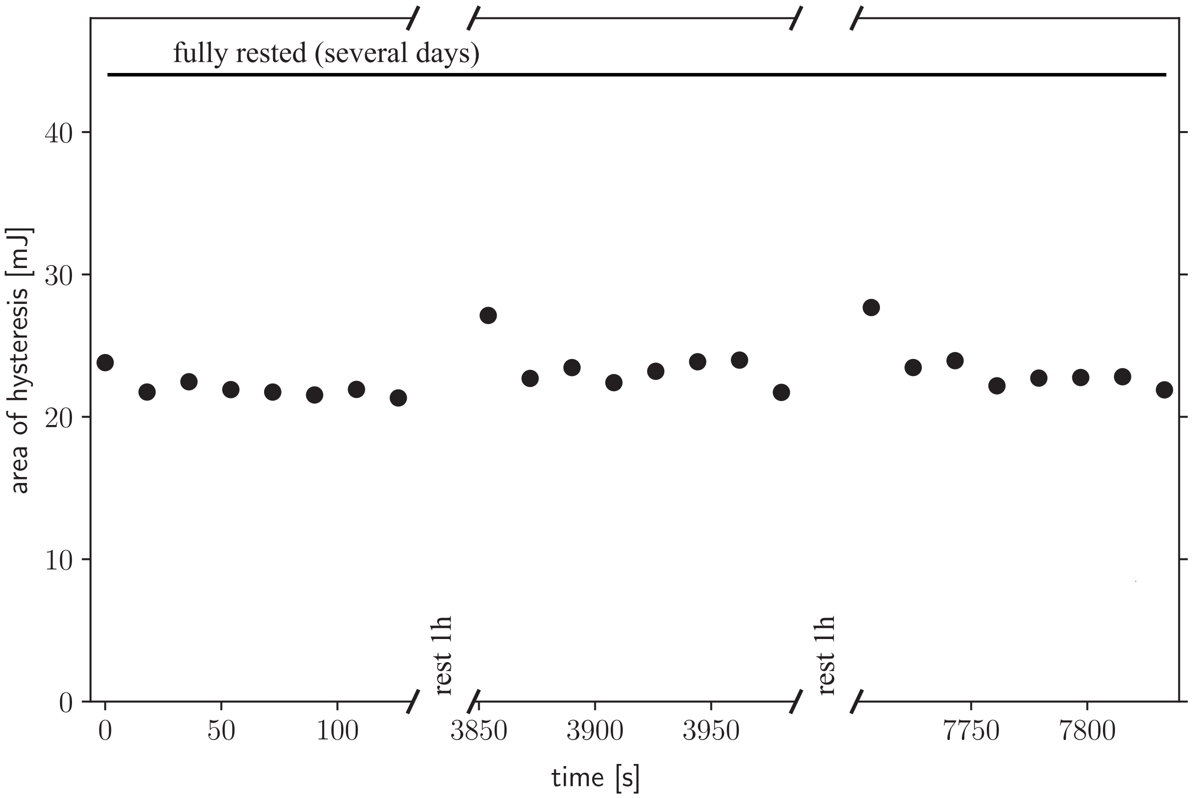

Figure S2: Dependence of Hysteresis Area on Cycling History. A sample encoded at $\mu_l = 1.55$ was subjected to intermittent cycling and rest periods. Each cycling phase consisted of eight consecutive cycles at 5 mm s^{-1}, followed by a one-hour rest period. The plotted course corresponds to an initially unrested sample.

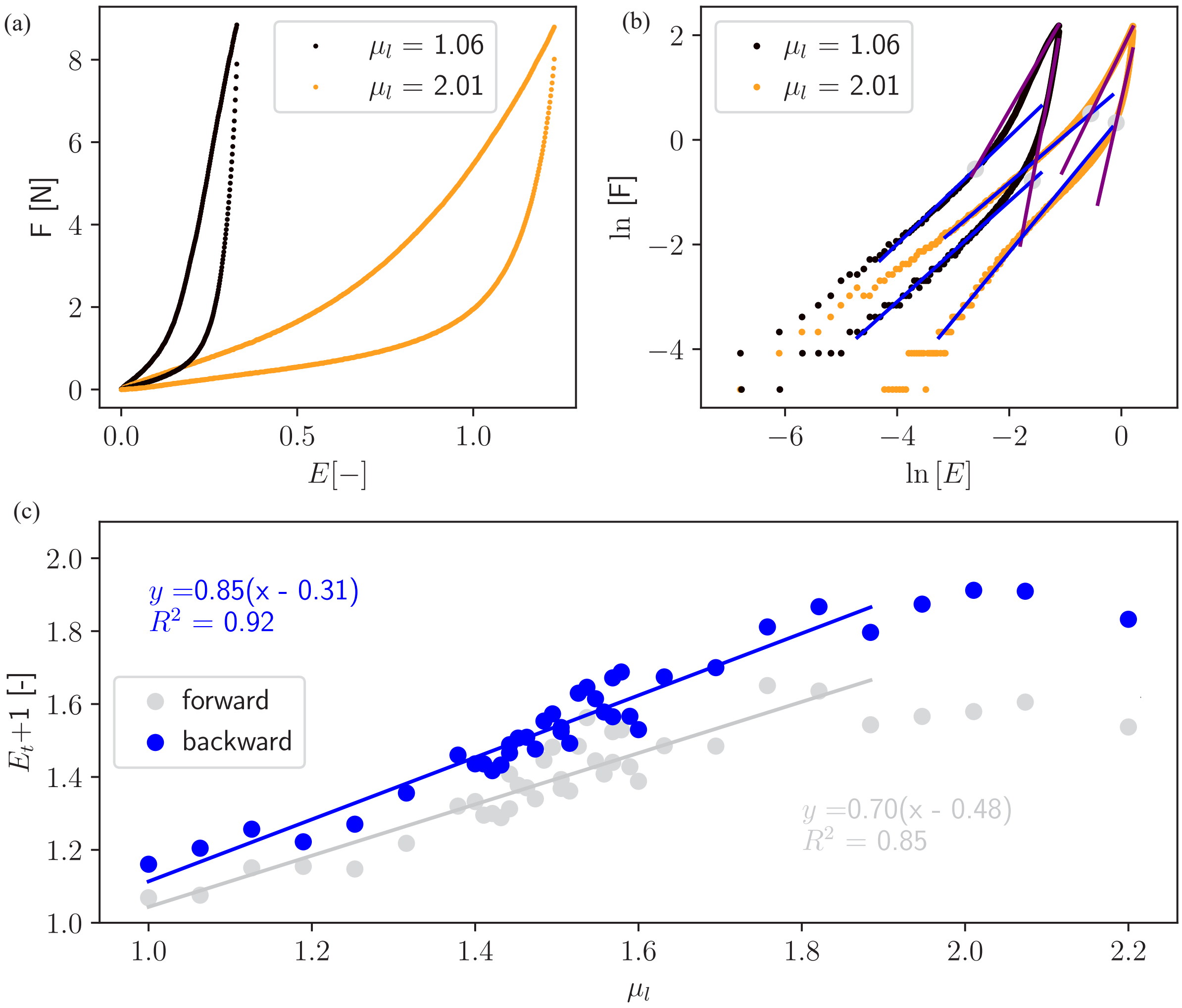

Figure S3: Forward and Backward Tensiometry. a) Force-strain plots of elastane encoded at small and large μ_l, including both loading and unloading courses. b) Log-log tensometry plots with linear fits for forward and backward scans, shown both above and below the inflection point E_t. c) The transitional strain as a function of μ_l for backward scans, with a linear regression line overlaid in blue. Forward scan data are shown in light grey for reference.

(a)

(b)

(c)

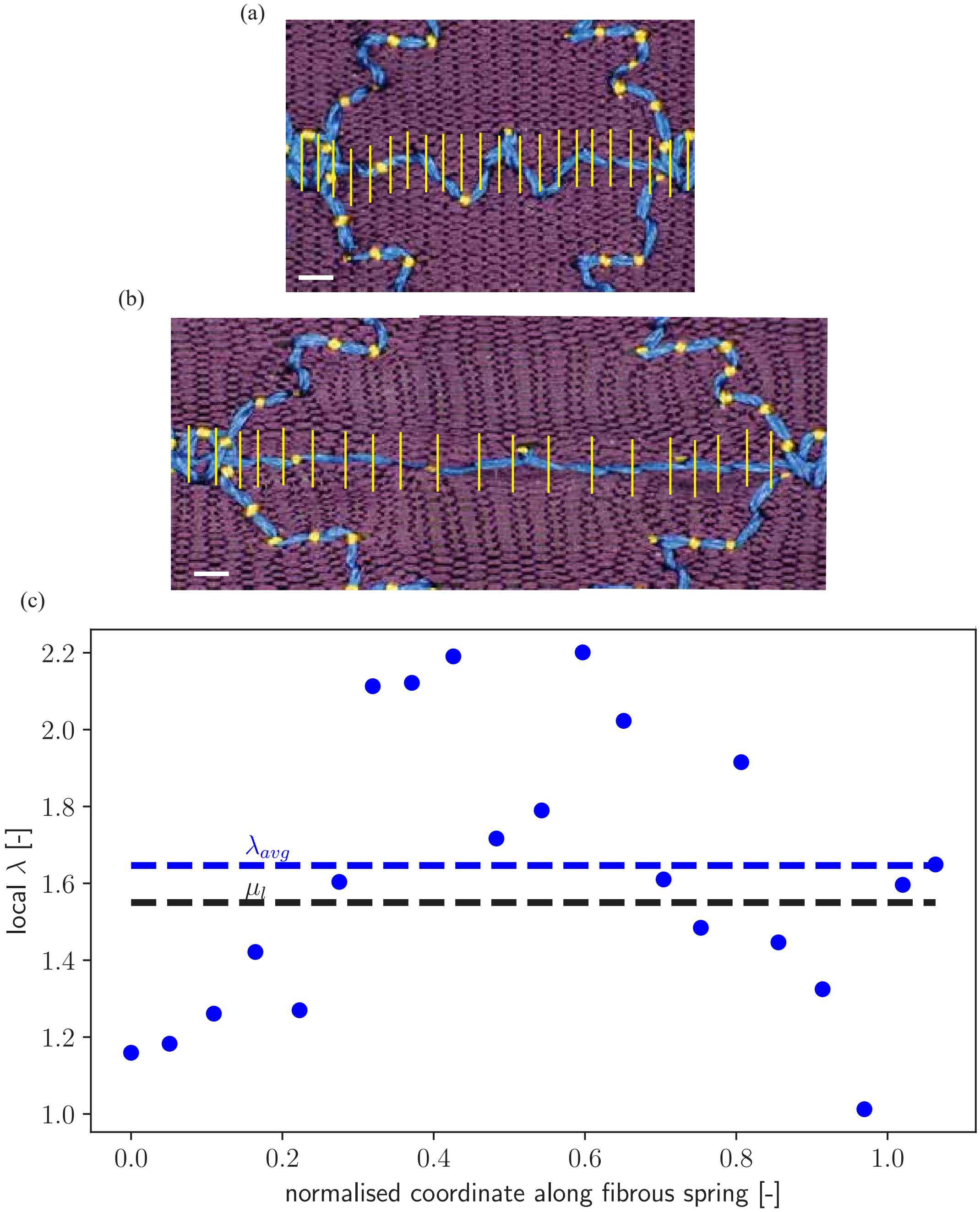

Figure S4: Local stretch in a fibrous spring. a)b) A fibrous spring encoded at $\mu_l = 1.55$ depicted a) rested and b) fully stretched. c) Local λ along the normalised coordinate of the fibrous spring. Values for global average stretch λ_{avg} and μ_l are added as horizontal dashed lines for visual reference. Scale bars: 1 mm.

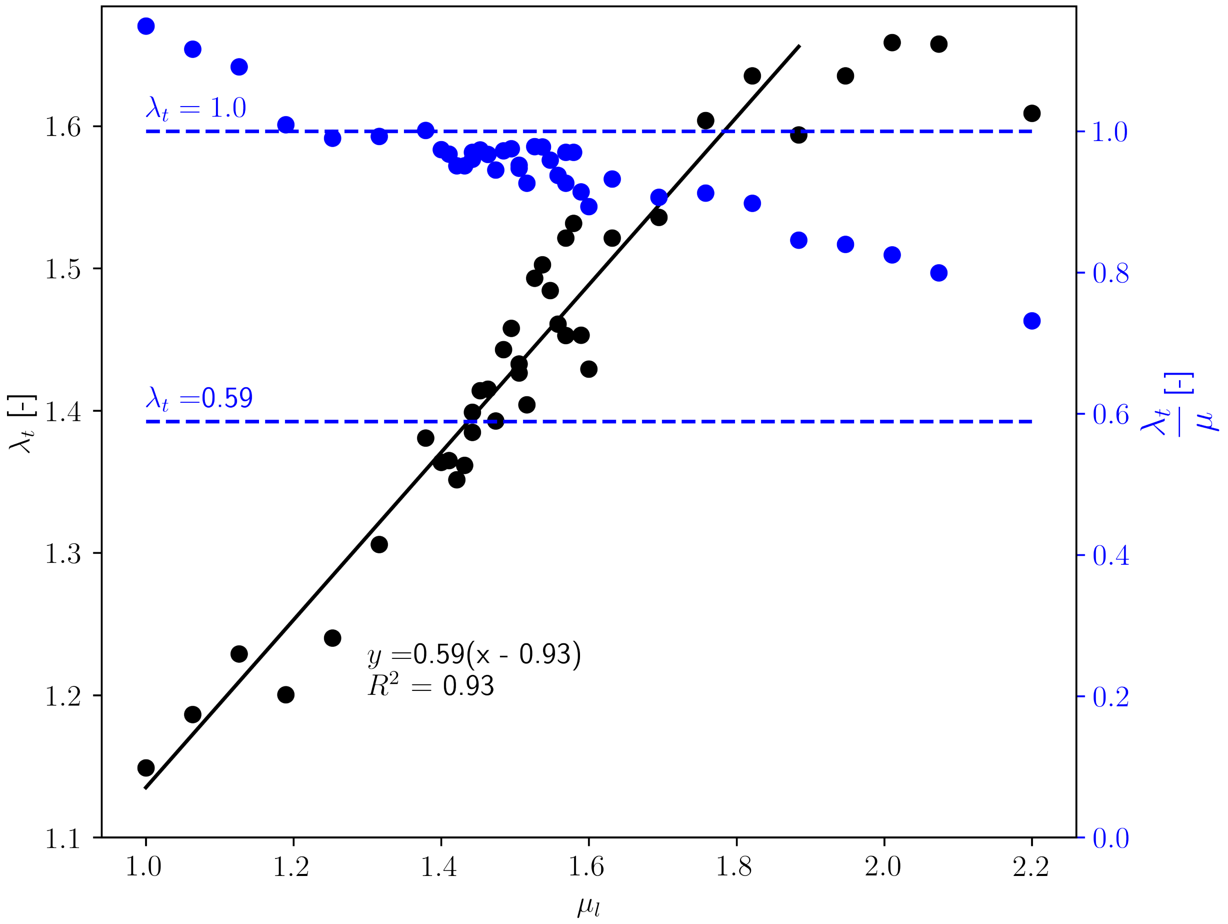

Figure S5: Ratiometric unpacking. Left axis: transitional stretch λ_t with respect to μ_l, including a fit for the stretch-referenced proportionality constant $\alpha_\lambda = 0.59$. Right axis: ratiometric unpacking parameter λ_t/μ_l with respect to μ_l. Blue dashed horizontal lines are guides for the eye for indicating limit values for λ_t/μ_l.

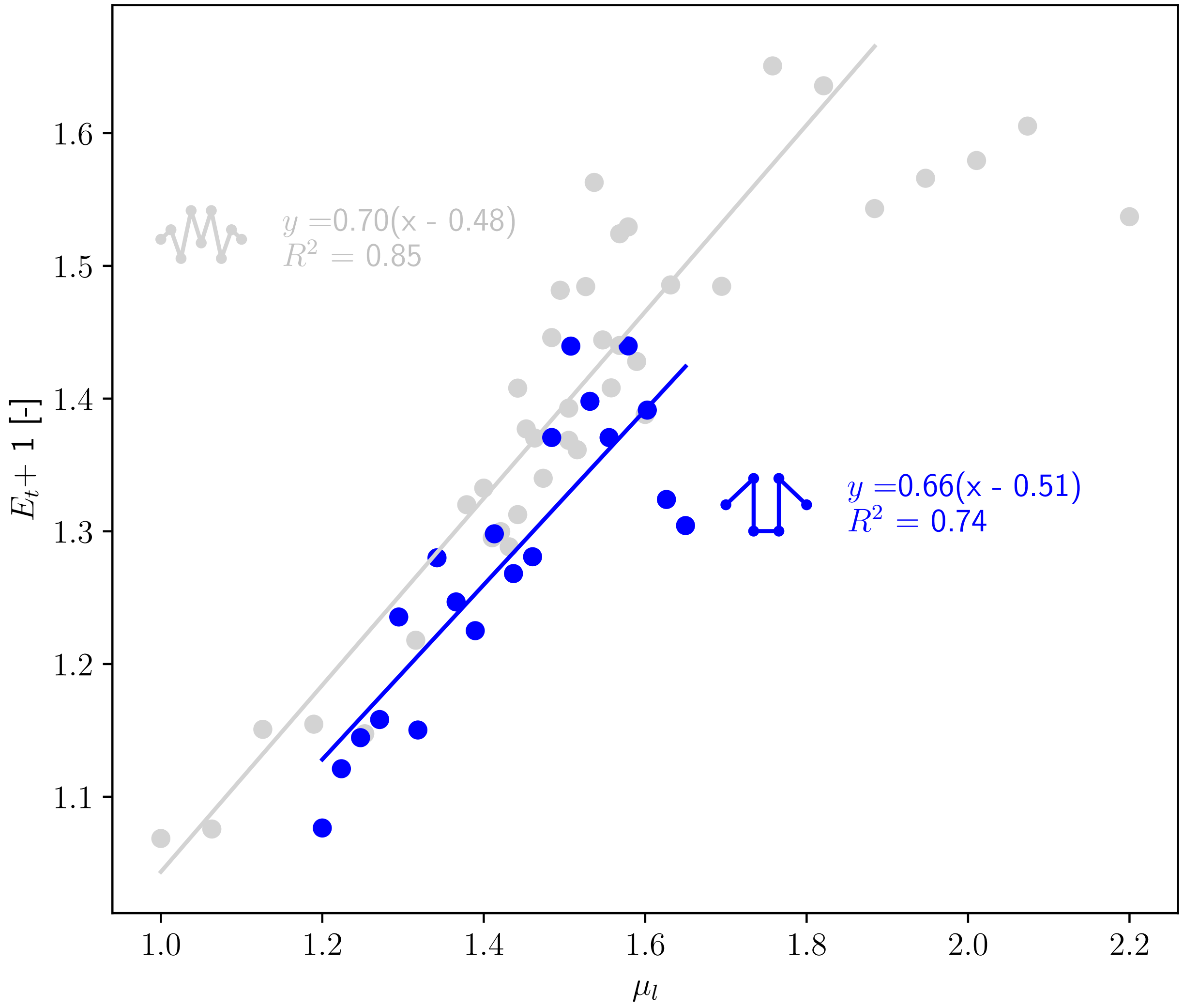

Figure S6: Pattern-Invariance of μ_l. The dependence of transitional strain on μ_l is shown for a rectangular fibrous spring pattern, with a linear regression line overlaid in blue. The zigzag pattern's corresponding data is in light grey for reference.

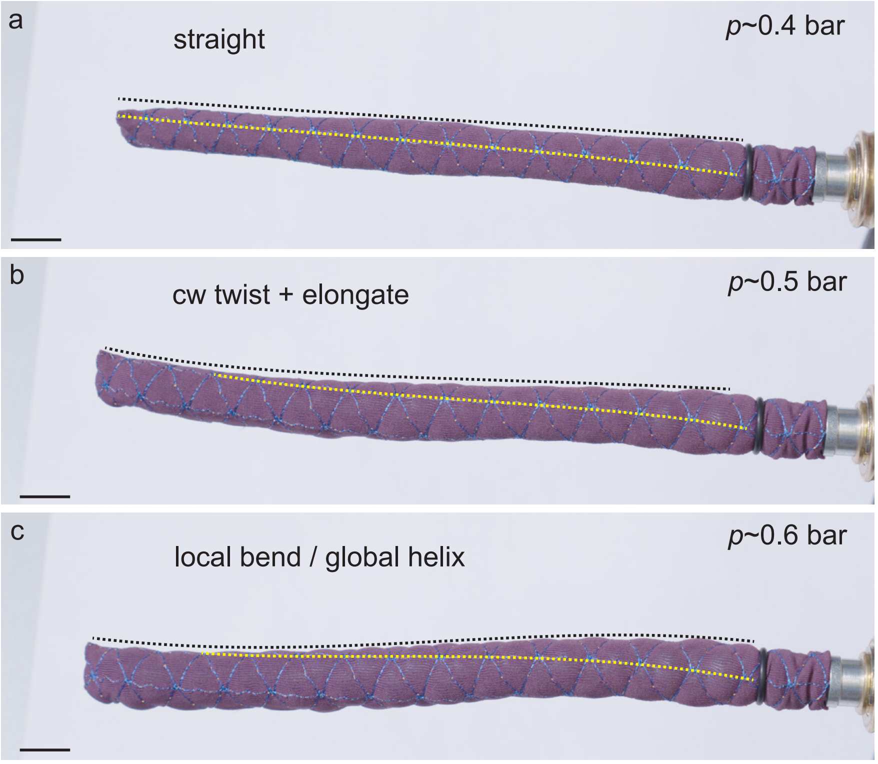

Figure S7: A restrictor bladder actuator with sequentially activated modes. (a) starting position at a low internal pressure, straight actuator. (b) Twist and elongation at medium internal pressure. (c) Local elongation and global twist at an increased pressure. Scale bars: 10 mm

Legend for additional supporting information

Movie S1, Supporting Information

Dynamic behaviour of the reported restrictor bladder actuators with different encoding patterns. In the first part of the movie, the restrictor's distal half was encoded for twist, while the basal region was encoded for minimal deformation. In the second part, the distal half of the actuator was programmed to twist and elongate. In the third part, the restrictor was encoded for uniform bending.

Movie S2, Supporting Information

A trimodal restrictor bladder actuator. At low pressures, the actuator twists and elongates. At higher pressures, the actuator bends locally, leading to global helical motion. The second half of the movie demonstrates over-pressurisation of the actuator, which demonstrates the resilience provided by the continous restrictor network to pressures beyond the working envelope.

Code Library S1, Supporting Information

Python library for generating embroidery stitch coordinates.

Code Library S2, Supporting Information

Python library for compiling the embroidery stitch coordinate patterns into an instrument-specific binary file, tested with the Pfaff Creative Icon embroidery machine.
